# A Recent Update on the Impact of Nano-Selenium on Plant Growth, Metabolism, and Stress Tolerance

**DOI:** 10.3390/plants12040853

**Published:** 2023-02-14

**Authors:** Ramkumar Samynathan, Baskar Venkidasamy, Karthikeyan Ramya, Pandiyan Muthuramalingam, Hyunsuk Shin, Pandy Saravana Kumari, Sivakumar Thangavel, Iyyakkannu Sivanesan

**Affiliations:** 1 R&D Division, Alchem Diagnostics, No. 1/1, Gokhale Street, Ram Nagar, Coimbatore 641009, India; 2 Department of Oral and Maxillofacial Surgery, Saveetha Institute of Medical and Technical Sciences (SIMATS), Saveetha Dental College and Hospitals, Saveetha University, Chennai 600077, India; 3 Department of Biotechnology, CMS College of Science and Commerce, Coimbatore 641049, India; 4 Division of Horticultural Science, College of Agriculture and Life Sciences, Gyeongsang National University, Jinju 52725, Republic of Korea; 5 Department of Microbiology, Rathnavel Subramaniam College of Arts and Science, Coimbatore 641402, India; 6 Post Graduate Department of Microbiology, Ayya Nadar Janaki Ammal College, Sivakasi 626124, India; 7 Department of Bioresources and Food Science, Institute of Natural Science and Agriculture, Konkuk University, Seoul 05029, Republic of Korea

**Keywords:** selenium nanoparticles, SeNPssynthesis, plant growth, stress tolerance, physiology, yield

## Abstract

Selenium (Se) is a microelement that plays an important nutrient role by influencing various physiological and biochemical traits in plants. It has been shown to stimulate plant metabolism, enhancing secondary metabolites and lowering abiotic and biotic stress in plants. Globally, the enormous applications of nanotechnology in the food and agricultural sectors have vastly expanded. Nanoselenium is more active than bulk materials, and various routes of synthesis of Se nanoparticles (Se-NPs) have been reported in which green synthesis using plants is more attractive due to a reduction in ecological issues and an increase in biological activities. The Se-NP-based biofortification is more significant because it increases plant stress tolerance and positively impacts their metabolism. Se-NPs can enhance plant resistance to various oxidative stresses, promote growth, enhance soil nutrient status, enhance plant antioxidant levels, and participate in the transpiration process. Additionally, they use a readily available, biodegradable reducing agent and are ecologically friendly. This review concentrates on notable information on the different modes of Se-NPs’ synthesis and characterization, their applications in plant growth, yield, and stress tolerance, and their influence on the metabolic process.

## 1. Introduction

Materials with a dimension smaller than 100 nm are referred to as nanomaterials (NMs). NMs have improved surface-to-volume proportions owing to their small size, which gives the man edge over their traditional equivalents. Since they are tiny in size and have an enhanced surface area, chemical composition, stability, and form, an aggregation of nanoparticles (NPs) has distinctive physicochemical properties: limited surface area, unusual surface structure, and heightened reactivity. Applying diverse NPs and nanomaterials (NMs) effectively improved crop plant nutrition compared to standard fertilizers. Additionally, compared to their bulkier counterparts, NPs less than 100 nm exhibit unique physicochemical, electrical, optical, and biological characteristics [1]. Due to their unique characteristics, such as their substantial surface-to-volume quotient, solubility, and multifunctionality, selenium nanoparticles (Se-NPs) are distinguishable from other materials.

The use of nanotechnology in creating nutraceuticals has many benefits, including enhancing the bioavailability of encapsulated bioactive natural compounds and promoting their controlled release and targeted delivery, both of which increase their biological efficacy, as reviewed in [2]. Additionally, NPs modify gene expression, which changes the molecular pathways of plants. Silver (Ag), gold (Au), ferric oxide (Fe_2_O_3_), zinc oxide (ZnO), selenium (Se), titanium dioxide (TiO_2_), aluminum oxide (Al_2_O_3_), silicon dioxide (SiO_2_), carbon NPs, nanotubes, nanowires, and quantum dots are examples of metal and non-metal oxides of NPs that play vital roles in plant germination, repair, and stress reduction. Applying nanotechnology in agricultural distribution to plant technologies holds the promise of targeted release of different types of macromolecules for increased immunity to plant diseases, efficient nutrient usage, and enriched plant development [3]. The synthesis of NPs in a unique, cost-efficient, eco-friendly way has been made possible using phyto-nanotechnology. The advantages of phyto-nanotechnology include scalability, biocompatibility, and the synthesis of NPs using a global solvent (water) as a reducing agent. Phyto-nanotechnology uses plants and their distinct plant parts, including roots, fruits, stems, seeds, and leaves, to create nanoparticles. Owing to their microscopic size and distinctive surface properties, nanoparticles are an alluring approach to regulating agricultural production systems [4,5], resulting in the recent focus on the impacts of NPs and NMs such as macro, micro, and nanocarrier-mediated fertilizers and plant-growth increasing NPs with unknown mechanisms, application concentration/rate, particle size, mode of action, and hazardous effects [6].

A forthcoming technology called nano-assisted agriculture has the potential to advance plant breeding and agricultural practices as well as increase tolerance to biotic and abiotic challenges [7]. Slow nutrient release from NPs may encourage plant development by maximizing nutrient supply and boosting the antioxidant defense system [8]. A new field, “plant nanoscience,” outlines the interplay between plants and nanotechnology. Nanosensors, nanopesticides, nanofertilizers, and nano-plant genetic engineering can all help to increase agricultural yields significantly.

Selenium (Se) is a non-metal or metalloid element that is present in a diverse range of forms, particularly as selenide [Se (-II)], elemental selenium [Se (0)], selenite [Se (IV)], or selenate [Se (VI)], all of which exist naturally, occurring in the environment, and accumulate in a variety of organisms [9]. The content of Se in soil has been in the range between 0.01 and 2 mg kg^−1^ [10]. Plant growth, biochemistry, and productivity changes caused by selenium are determined by many parameters, notably Se source, experimental techniques, growth rate, and variety of plant species. In a limited extent of doses (below 10 mg L^−1^), application of Se may increase growth rates, enhance nutritional content, not alter nuclear transcription profiles, increase photosynthesis efficacy, induce antioxidant profile, alter hormonal balances, enhance primary and secondary metabolism, and enhance plant accustomed to unfavorable environmental conditions [11]. Typically, selenium-rich agricultural products are regarded as a practical and efficient strategy to raise selenium levels in human bodies [12]. Se-NPs have various advantages, including low toxicity, high degradability, and excellent anticancer, antimicrobial, and antiviral properties [13,14].

The Se accumulated within plants is greatly impacted by varied circumstances, including the levels of selenium in the soil ecosystem and the concentrations plant species uptake themselves [14,15]. Plant-mediated generation of Se-NPs is superior to conventional biosynthesis strategies. Natural stabilizing and reducing agents found in plants can produce Se-NPs in an economically feasible and eco-friendly safe manner. Among other nanomaterials, plant-derived Se-NPs have shown promise as potent antibacterial and antioxidant agents to lessen the detrimental impacts of plant diseases in many crops [16]. Se NPs have shown promising clinical benefits in a range of oxidative stress and inflammation-mediated diseases, such as diabetes, cancer, nephropathy, and arthritis [17].

The biocompatibility, bioavailability, low toxicity, biodegradability, and environmental friendliness of Se-NPs derived from plants have all been demonstrated to be quite high. This is due to the presence in the plant extract of secondary metabolites such as phenols, tannin sesquiterpenes, cinnamic acid, and monoterpenes that serve as reducing agents and stabilize capping during the production of Se-NPs and sustain them as environmentally friendly [18]. Depending on the synthesis conditions, their wide use in biomedical applications is determined by their size, structure, and biochemical characteristics [18]. In this review, we highlighted the different routes of the synthesis of Se-NPs and their imminent applications in plant growth and development. Further, their role in the regulation of plant metabolites was discussed in detail.

## 2. Modes of SeNP Synthesis

**Figure 1:**
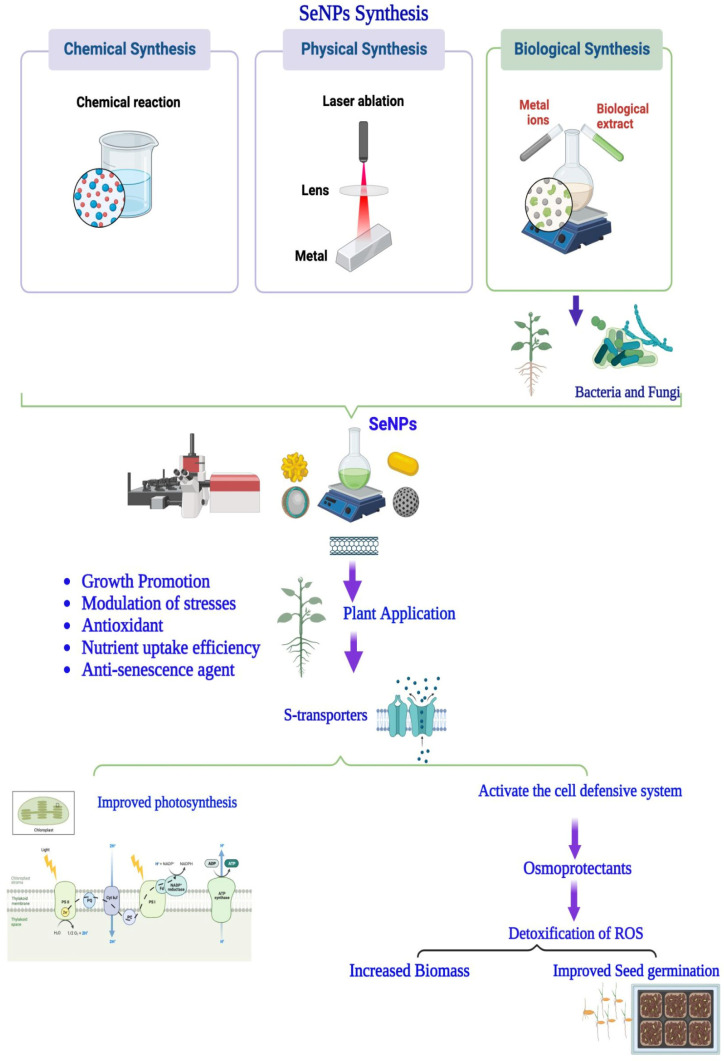
An overview of various modes of synthesis and application of SeNPs in plants.

NPs have emerged recently due to their distinctive qualities, including morphological propensity, chemical reactivity, competitive binding ability, and optical activity. SeNPs are synthesized by three basic methodologies, namely physical, chemical, and biological approaches (Figure 1).

Several investigations have suggested physical means of synthesizing Se-NPs by employing pulsed laser ablation and ultrasound-based synthesis, vapor deposition, and hydrothermal and solvothermal techniques for synthesizing Se-NPs [19]. Gudkov et al. [20] proposed a method for producing Se-NPs in the zero-valent state. Se-NPs were formed by laser ablation of Se in water utilizing a fiberytterbium laser at a wavelength of (b/w 1060 and 1070 nm), where the repetition of the pulse rate is 20 kHz (Kilohertz). The fundamental particle mass component altered from a size of 800 nm to less than 100 nm due to increased laser fragmentation durations. The resulting SeNPs were mono-dispersed in both mass and size.

Se-NPs are synthesized chemically from precursors that are inorganic forms of selenium. To prevent nanoparticle aggregation, reducing agents such as glucose, ascorbic acid, fructose, cysteine, glutathione, sodium metasulfate, and the ionic liquid 1-ethyl-3-methylimidazolium thiocyanate have been employed in Se-NP synthesis. Panahi-Kalamuei et al. [21] used chemical substances, which include SDS (Sodium dodecyl sulfate), CTAB (Cetyl Trimethyl Ammonium Bromide), and polyethylene glycol (PEG 600) to synthesize Se-NPs. The initial material was SeCl_4_ (Selenium tetrachloride), which, when dissolved in water, creates selenious acid and is reduced with hydrazine hydrate. Se^4+^/Se has a reduction potential of 0.74 eV, while N_2_H_4_H_2_O/N_2_ (Hydrazine Hydrate/Nitrogen Gas) has a reduction potential of 1.16 eV. Ragavan et al. [22] exploited sodium selenite and ascorbic acid to produce Se-NPs by a precipitation process, and the physical properties of Se-NPs were characterized by scanning electronic microscopy (SEM). The size of the SeNPs ranged between 9.09 mm to 9.07 mm. The chemical properties of the Se-NPs analyzed with the Energy Dispersive X-Ray Analysis (EDAX) spectrum were recorded as two peaks between 1.6 Kev and 10.8 Kev. The diffraction peaks of the XRD (X-ray diffraction) include 22.0158°, 6.9573°, 2.7332°, and the Fourier-transform infrared spectroscopy (FTIR) spectrum in the region of 4000 to 400 cm^−1^, and O-H stretch, free hydroxyl-C-H stretch, H-C-H stretch = C-O bend, and C-O stretch bands were recorded at 3441 cm^−1^, 2920 cm^−1^, 2858 cm^−1^, 1625 cm^−1^, 1537 cm^−1^, 1324 cm^−1^, 1025 cm^−1^, and 1032 cm^−1^, which are coupled to each another.

El-ghazaly et al. [23] produced spherical Se-NPs around 13 nm in size in an ice-cold solution using SeO_2_ as the precursor and polyvinylpyrrolidone (PVP) as the stabilizing agent and KBH_4_ as the reducing compound. The presence of orange color suggested the creation of Se-NPs, and according to a transmission electron microscopy (TEM) study, the particle shape and size were 10 nm in diameter. Vahdati et al. [24] observed a distinctive Ultraviloiet (UV) absorption peak at 265 nm, comparable with prior reports but with considerably smaller sizes in the region of 35–45 nm as compared to polyvinyl acetate or chitosan.

Se-NPs are created from selenate using a powerful reducing cum stabilizing agent, ascorbic acid (AA). The typical surface plasmon absorption peak at 296 nm in the UV-vis spectrum verified the synthesis of nanosized sodium selenate (Na_2_SeO_4_) at 10 mm using 1.5% AA. The TEM results of the produced Se-NPs revealed that Se-NPs are generally spherical with a diameter of 33.4 nm. The XRD pattern validated that Se-NPs are crystalline and have a particle size of 42.92 nm [25].

El Lateef Gharib et al.’s [25] study compared the treated supplementation of Na_2_SeO_4_ and Se-NPs up to 25 M and untreated plants and recorded that entire plants treated with Se-NPs observed a significant increase in foliar growth, including different plant parts along with total leaf area cm^2^/plant. With Na_2_SeO_4_ and Se-NP application, particularly at 6.25 M concentration, the biochemical observations of the total content of photosynthetic pigments (TPP), carbohydrates (TC), soluble proteins (TSP), and other minerals in leaves were improved. In these studies, the cowpea leaves were treated with Se-NPs at 6.25 M, which elevated levels of the growth gibberellic acid (GA_3_), indole acetic acid (IAA), and cytokines (CKs), then with Na_2_SeO_4_ at 6.25 M. This explains how plants treated with Se-NPs and Na_2_SeO_4_ had higher growth parameters and heavier seeds than untreated plants.

The microbially synthesized Se-NPs were examined using dynamic light scattering (DLS), which showed that the size varied. Particle size ranged between 120 and 260 nm, with an average diameter range of 10–55 nm. Se-NPs zeta potential analysis revealed a negative potential (32.42 mV) in ddH_2_O (double distilled water), and TEM analysis revealed that all Se-NPs were spherical NPs [26].

*Allium sativum* L. clove extract-based Se-NPs were validated using the UV-visible spectrum between 200 and 800 nm, demonstrating and analyzing the bio-fabricated Se-NPs’ peak between 200 and 400 nm. The maximal characterization peaks of selenium measured using energy dispersive X-rays ranged from 2.5 to 3.5 Kev. SEM studies on green-synthesized Se-NP particles revealed that they are spherical, cylindrical, or rectangular, with most being 40–100 nm in size [27]. The production of Se-NPs via green nanotechnology, notably employing microbes and plants or their associated by-products (microbially synthesized proteins/enzymes, secondary metabolites, and lipids), as well as diverse biotechnological processes, is indeed gaining popularity [13]. These strategies are eco-friendly, cost-effective, eliminate hazardous and harsh chemicals, and use subtle energy. Plants serve as both stabilizing and reducing agents, allowing for fine control of nanoparticle size and distribution. Due to the massive importance of the biological generation of Se-NPs, currently, several researchers have examined the use of plant parts (Table 1). Numerous studies have investigated the photosynthesis of Se-NPs utilizing raisin extract as a capping, stabilizing, and reducing agent. The adequate concentration of plant extract employed for it and the physicochemical properties characterizing the diverse surface plasmon resonance bands affect Se-NPs’ biocompatibility, stability, and adaptability, as reviewed in [16]. Se-NPs biosynthesis encompasses the use of living microorganisms and plants to convert sodium selenite salt to Se-NPs, including the fungus *Mariannaea* for Se-NP biogenesis [28]. Biosynthetic Se-NPs have many advantages over both organic and inorganic selenium compounds. As a result, the biological synthesis of Se-NPs is preferred for increased biocompatibility and stability. In Se-NPs synthesized with an extract from the *Vitis vinifera* (raisin), Sharma et al. [29] found the least zeta potential values equal to 36 V, followed by Java tea extract (34.9 V) [30], and the cocoa bean shell was 28.6 V [31]. Joshi et al. [32] synthesized the antifungal activity of mycogenic Se-NPs and, through SEM-EDS (Scanning Electron Microscopy—Energy Dispersive X-ray Spectroscopy analysis) of bioactive Se-NPs revealed that they are spherical in size (60.48 nm to 123.16 nm).

The biosynthesis of Se-NPs has also been found to involve a wide variety of bacterial strains, including *Klebsiella pneumoniae* and *Pseudomonas aeruginosa,* as reviewed in [16]. According to Seliem et al. [33], Se-NPs of 50–100 nm size were produced using *Lactobacillus casei*, and the biosynthesized Se-NPs improved the physiological and biochemical profiles of two sensitive chrysanthemum cultivars—namely, *Sensuous* and *Francofone* under heat stress by increasing antioxidant enzyme activity.

The Se-NPs synthesized using glutathione as a reducing agent exhibit a size of 12.7 nm. The resultant biosynthesized Se-NPs showed inhibitory activity against the *Sclerospora graminicola* and *Alternaria solani* [34]. According to Khalifa and Sameer [35], Se-NPs inhibited the mycelia growth of fungi *Penicillium digitatum* to 5.56% at 100 ppm, and 85.22% at 500 ppm concentration, which is the cause of green mold disease of orange fruit. The *Sclerospora graminicola* biosynthesized Se-NPs inhibit vegetative growth, sporulation, spore viability, and proliferation [36]. El Badri et al. [37] synthesized biogenic Se-NPs (bio Se-NPs) with sizes ranging from 120 to 260 nm, with a mean size of 167 nm. Six species of *Trichoderma* were examined for their ability to produce Se-NPs, including *T. asperellum*, *T. harzianum*, *T. atroviride*, *T. virens*, *T. longibrachiatum*, and *T. brevicompactum*. These Se-NPs were then used to control the severity of the *Sclerospora graminicola*-induced downy mildew disease in pearl millet. In the UV-visible spectral range from 200 and 400 nm, Se-NP solutions exhibited the highest absorption [36]. Employing leaf extract from the *Pelargonium zonale* against *P. digitatum*, Se-NPs demonstrated remarkable potential. *P. digitatum*, a plant pathogen, is a significant cause of the post-harvest fungi known as green mold in citrus [38].

**Table 1:** Green synthesis of Se-NPs using different plant extracts and their characterization methods.

Method of Synthesis	Name of Plant Source	Size	Shape	Operational Parameters	References
Green-synthesis	*Vitis vinifera* Extract	3–18 nm	Spherical	10 mL of Extract added with 4 × 10^−5^ M selenous acid for refluxed for 15 min and centrifuged at 15,000× *g* rpm	[29]
	*A. sativum*	48–87 nm	Spherical	Garlic extract (5 mL) mixed with 20 mM Na_2_SeO_3_ solution (50 mL) and stirred at 150 rpm at 60 °C.	[39]
	*Clausena dentate* leaf extracts	46.32–78.88 nm	Spherical	10 g of *C. dentata* leaf powder with 100 mL of DH_2_O boiled at 60 °C for 5 min. This broth (12 mL) was added to 1 mM aqueous selenium powder (88 mL) for the synthesis of Se-NPs	[40]
	*Crataegus monogyna*	113 nm	Spherical	0.01M Na_2_SeO_3_ was mixed with 2 mg mL^−1^ hawthorn fruits lyophilized powder for 12 hrs and dialyzed (MWCO 8000–14,000) for 48 hrs.	[41]
	*Emblica officinalis*	20 to 60 nm	Spherical	2 mL of aqueous fruit extract of *E. officinalis* was added with 10 mL of 10 mM Na_2_SeO_3_ under stirring. The mixture was allowed in dark conditions at 27 ± 2 °C and 120 rpm for 24 hrs.	[42]
	*Catharanthus roseus* and *Peltophorumpterocarpum* flowers	23.2 nm and 30.44 nm	Spherical	10 gm of the flowers was added with 90 mL of 10 mM aqueous solution of Na_2_SeO_4_ and incubated at 250 rpm at 36 °C for 7 days	[43]
	*Withania somnifera* leaves extract	15 nm	Crystalline	One hundred milli liters of plant extract was mixed with 50 mM selenious acid and stirred and washed with distilled water and acetone at overnight	[44]
	*Aloe vera* leaf extract (ALE) Fabricated with Se-NPs	50 nm	Spherical fabricated Se-NPs	20 mL of culture filtrates, cell lysate, and crude cell wall from six *Trichoderma* spp was added with 70 mL of sterile distilled water containing 25 mM Na_2_SeO_3_ and stored 28 ± 1 °C on a shaker at 150 rpm for 6 days	[45]
	*Zingiber officinale*	100 to 150 nm	Spherical	1% ginger extract was added to 10 mM Na_2_SeO_4_ solution (9:1 ratio) with pH 9 at 37 °C for 75 hrs at 130 rpm	[46]
	Wheat (*Triticum aestivum* L.)	140 ± 10 nm	Spherical	**Biosynthesized SeNPs**: *Rahnella aquatilis* HX2 cell broth filtered in Na_2_SeO_3_ at 5 mM for 48 hrs at 28 °C then centrifuged at 8000× *g* for 30 min and washed with 1 M NaOH for 20 min in a boiling water bath.	[47]
	*Vigna unguiculata* L.	33.4 nm	Spherical	10 mM of aq. solution of 10 mM Na_2_SeO_4_ added to the ascorbic acid powder 1.5% (*w*/*v*) under stirring at room temperature for 15 min.	[25]
	*Cyamopsis tetragonoloba*	50–150 nm	Oval	700 mg of Na_2_SeO_3_ in 50 mL of distilled water for 20 min, added with 50 mL of ascorbic acid solution	[22]
	*Raphanus sativus* var. sativus, *Eruca sativa*, *Solanum melongena*, *Cucumis sativus* and *Capsicum annuum*.	100 nm	X-ray diffractograms	Laser ablation processes of solid Se then fiber ytterbium laser (1060 nm and 1070 nm), pulse rate 20 KHz in 80 nanoseconds in 20 W	[48]
	*Brassica napus*	10–55 nm	Spherical	1%*Comamonastestosteroni*S44 culture added with 10 mM Na_2_SeO_3_ for 72 hrs. Then, 2 M NaOH solution was added to this under stirring	[26]
	*Allium sativum*	40 to 100 nm	Spherical	*Cassia auriculata* in fine powder added with 100 mL of 10 mM Na_2_SeO_3_ at a concentration (10 × 10^−3^ M) under magnetic stirring, then incubated at 37 °C	[49]

## 3. Mode of Action

The diversification of selenium, which occurs in several environmental compartments in various forms, impacts its accessibility and distribution based on numerous characteristics, including pH, natural organic matter (NOM), microbial activity, redox potential, ionic elements, soil quality, temperature, and moisture [50]. Plant species, age, and selenium availability all affect how toxic selenium is to plants. Young plants are substantially more susceptible to Se toxicity than adult ones, whereas SeO_3_^2−^ is a more phytotoxic form of SeO_4_^2−^ [51]. Variations exist across plant species in Se absorption and accumulation and the production of volatile Se-compounds to prevent Se toxicity. Plants are divided into three types based on their ability to absorb, use, and accumulate Se. The primary Se accumulators, which contain 1000 µg Se g^−1^ DW, are followed by secondary Se accumulators, which contain 100 to 1000 µg Se g^−1^ DW, and non-accumulators, which contain less than 100 µg Se g^−1^ DW, as reviewed in [51]. Many studies have suggested that Se participates in upregulating antioxidant defense in hyperaccumulator species, where enzymatic and non-enzymatic antioxidants and phytohormones, such as jasmonic acid (JA), salicylic acid (SA), and ethylene (ET), play crucial roles in Se tolerance [52,53].

Plants take up selenium from the soil, and the Se-containing plants can be split into two categories: first, those with selenium concentrations equivalent to those present in the soil, and then those with selenium in significantly greater amounts than the concentrations present in the soil [54].

Despite the fact that there is still much to learn about this phenomenon, several directions have been suggested to investigate Se-NP absorption and uptake into plant systems [47]. The plant’s cell wall prevents external factors, such as Se-NPs, from entering the plant’s cell walls. Wang et al. [55] disclosed that Se-NPs could enter the plasma membrane and cross a cell wall. Se-NPs may adhere to plant roots and impact a plant’s chemical and physical absorption. The most universally acknowledged theory is that NPs are absorbed intra- and extracellularly via the tissues till they attain the xylem [11].

Selenite most probably enters the plant root via a phosphate transport channel, a metabolically energetic process. Selenite is converted to selenide and subsequently integrated into selenocysteine during the Se assimilation process (SeCys). Zhu et al. [56] investigated selenium and iodine intake by spinach (*Spinacia oleracea* L.) dispersed in a liquid and proposed that the cell wall performs as a physical blockade; moreover, it possesses pores with diameters of 5 to 20 nm where the NPs smaller than this can enter.

White and Broadley [57] stated that selenocysteine (SeCys) most likely occurs in the cytosol of the cell, chloroplasts, and mitochondria, and therein SeCys is transformed into selenomethionine (SeMet). It [58] was hypothesized that in the transfer of nanoparticulate materials to plants was also conceivable that nanoparticles larger than 20 nm widen the pores, causing holes through which endocytosis or transmembrane proteins or ionic channels could enter. Earlier research by Domokos-Szabolcsy et al. [59] showed that tobacco could absorb Se-NPs to root and callus cultures, and significant effects of Se-NPs in plant tissue culture varied from selenate. The uptake of Se-NPs is not simply based on cell wall pore diameter; additional mechanisms might well be implicated. According to Schiavon and Pilon-Smits [60], selenate is taken into the cell via a sulphate transporter in the root plasma membrane and subsequently transformed into Se-amino acids in plant metabolism.

Se-NPs may also be absorbed by plants, which then convert them into selenite and selenate in the roots and shoots, proving that Se-NPs are bioavailable to plants. The twenty-fold increased uptake rate of SeCys and SeMet by wheat and canola is greater than selenate or selenite, indicating that plants likely absorb organic forms of Se through amino acid permease, as reviewed in [47]. Hu et al. [47] demonstrated that after being absorbed by wheat roots, Se-NPs were promptly oxidized to Se (IV) and transformed into organic forms of either selenocysteine (SeCys_2_) or se-methyl-selenocysteine (MeSeCys), or selenomethionine (SeMet). Absorption and bio-transformation experiments on chemically and biologically synthesized 5 M nSe treated wheat seedlings discovered that nSe was absorbed by root aquaporins, and thereby SeMet and SeIV were accumulated in roots [47]. Aquaporins inhibitor prevented wheat roots from absorbing chemically synthesized Se-NPs (Che Se-NPs) and biologically produced Se-NPs (Bio Se-NPs) by 92.5 and 93.4%, respectively. Wheat roots absorbed Se-NPs at 1.8 and 2.2 times greater rates than 140 and 240 nm [47].

## 4. Impact of Se-NPs on Growth and Physiology

The impact of NPs varies, subject to the plant type, physiochemical characteristics, and application concentration. Different NPs affect the plant to improve its physiology, germination rate, and biomass productivity. Numerous NPs have distinct physical and chemical characteristics that make it simple for them to penetrate plant cells and can change their metabolism through a number of interactions to affect plant growth and development, which activates the capacity to withstand stressful circumstances [61].

In accordance with a number of research studies, a foliar supply of metal NPs significantly increases the amount of chlorophyll in plants, allowing them to produce more complexes for light harvesting in order to increase the absorption of light energy [62]. The impacts of NPs have also been thoroughly studied by looking at how they affect chlorophyll and photosynthetic efficiency and growth inhibition. Se-NPs operate as catalysts and strengthen plants’ antioxidant defense mechanisms, improving their ability to withstand biotic stress as reviewed in [16]. Chlorophyll a and b contents are minimized by tannic acid-capped Se-NPs at 20 and 80 mg L^−1^, respectively. This occurs as a result of the reactive oxygen species (ROS) produced by biogenic Se-NPs, which raise hydrogen peroxide (H_2_O_2_) and malondialdehyde (MDA) while lowering Peroxidase (POD) and other antioxidant defense enzymes [63]. Mozafariyan et al. [64] reported that when compared to control plants, 5 g of Se enhanced root growth and relative water content in hot pepper plants by 13%.

The vital roles of Se include stimulating plant growth by enhancing glucose metabolism, recovering chloroplast ultrastructure, accelerating chlorophyll biosynthesis, and preventing chlorophyll degradation [65]. According to Djanaguiraman et al. [66], Se-NPs decrease high-temperature mediated stress by boosting seed yield, pollen germination, percentage of seed setting, photosynthetic rate, and lowering oxidative stress. Cowpea seeds were treated with foliar applications of Na_2_SeO_4_ or Se-NPs at 6.25 and 12.5 M, which resulted in higher levels of Total Carbohydrate (TC) and Crude Protein (CP) than controls at 105 days after planting. The Se-NP supplementation at 6.25 M concentration, followed by Na_2_SeO_4_ at the identical concentration, produced remarkable mineral content values for N, P, K, Ca, S, and Mg in dry cowpea leaves [25]. According to Al-Deriny et al. [67], Se-NPs at low concentrations cause changes in jasmonic acid, salicylic acid, and ethylene, and their signaling causes changes in metabolism along with the expression of defensive genes. *Trichoderma asperelleum* + Se-NP application enhanced plant height (24.9 cm), tiller count (3.40 tillers/seedling), and content of chlorophyll (3.76 mg g^−1^) in pearl millet seedlings, according to Nandini et al. [36]. Se-NP concentrations between 50 and 100 mg kg^−1^ greatly boosted the root system (>40%) and organogenesis [68]. Additionally, Se-NPs support organogenesis and root formation. A trace level of Se has been demonstrated to promote growth in *Brassica oleracea*, potato, lettuce, and ryegrass plants [69]. Dai et al. [70] examined the beneficial effects of Se in *Brassica campestris* sp. *Pekinensis* grew under two different Se and Zinc accumulation treatments, revealing that Superoxide dismutases (SOD), POD, Catalase (CAT), Glutathione peroxidase (GR), Ascorbate peroxidase (APX), and proline concentration were enhanced when Se was present.

El-Kinany et al. [71] revealed that applying foliar nSe (25 and 50 mg L^−1^) with the surfactant tween 80 (0.005%) twice every 15 days enhanced yield and ascorbic content in NaCl salt-stressed coriander (*Coriandrum sativum* L.) plants. Additionally, Alves et al. [72] observed that when the treatment of 1.0 M of selenite or selenate was added to cadmium (Cd) toxicity (0.5 mM CdCl_2_)-stressed tomato plants, they exhibited an increase in photosynthesis and biomass. While Se and Zn (10, 20, and 40 mg L^−1^) were applied together, wheat’s Cd-induced loss in photosynthesis, stem growth, and pigmentation was significantly reduced [73]. Maize growth and chlorophyll content were raised by selenium treatment (20 mg L^−1^) under increased saline stress, which causes high MDA and H_2_O_2_ [74]. APX, SOD, and CAT were upregulated by 44%, 56%, and 57%, respectively. Rady et al. [75] found that tomatoes treated with 40 M of Se had enhanced drought tolerance.

Xu et al. [76] demonstrated a rise in CAT levels following Se supplementation in rice under Cd toxicity stress, indicating that Se has a beneficial effect in improving plant resistance to stress. Behbahani et al. [11] investigated nSe (0, 1, 4, 10, 30, and 50 mg L^−1^) or bulk (selenate) treatments in bitter melon seedlings and concluded that they dramatically increased to an average of 52% in leaf nitrate reductase activity compared to the control. Micro-measurement analyses at 1 mg L^−1^ induced the growth of primary and secondary tissues. However, the nSe-applied seedlings had considerably enhanced Pheylalanine ammonia lyase (PAL) activity in the roots and leaf-soluble phenols compared to the control. The impact of selenium on *Vicia faba* L. minor roots exposed to lead (Pb) stress was analyzed by examining root growth, root viability, and antioxidant enzyme activity, according to Mroczek-Zdyrska and Wójcik [77]. Molnár et al. [78] reported that selenite sensitivity in *Arabidopsis* resulted in plant alterations such as the opening of stomatal openings, aggregation of callose, severe oxidative stress, and moderate nitrosative changes. These changes were also associated with reduced stomatal density. Cowpea (*Vigna unguiculata* L) plants that adapted to foliar sprays of either Na_2_SeO_4_ up to 25 M or Se-NPs up to 50 M concentration were investigated by El Lateef Gharib et al. [25]. They found that both nanocomposite treatments significantly increased the Chlorophyll a and b and carotenoid levels more than the control till 75 days after sowing. The increment in chlorophyll and carotenoid content in the cowpea leaf was found with Se-NPs at 6.25 M concentrations, followed by Na_2_SeO_4_ at 6.25 M concentrations. According to Skalickova et al. [79], applying Se-NPs in agricultural practice protects against salt and high temperatures. Se-NPs and SeO_3_^2^ ameliorate *Brassica napus* L. growth under Cd toxicity stress due to Se-NPs. However, Yu et al. [80] found that in *Brassica chinensis*, 10 M of either SeO_3_^2−^ or SeO_4_^2−^ improved antioxidant defense and prevented the formation of H_2_O_2_ and MDA. By limiting the development of NADPH (Nicotinamide adenine dinucleotide phosphate) oxidases (*BnaRBOHD1*, *BnaRBOHC*, and *BnaRBOHF1*) and glycolate oxidase (*BnaGLO*), the form of Se-NPs reduced Cd-induced reactive oxygen species generation, hence reducing oxidative protein and membrane lipid degradation.

Plant growth and development, K^+^ content, and photosynthetic rate all increased with one mM Se, while the ratio of Na^+^ decreased. Additionally, by lowering oxidative damage caused by high MDA and H_2_O_2_ under enhanced salinity stress, selenium treatment (20 mg L^−1^) enhanced maize growth and chlorophyll level [74]. Se-NPs also increased resistance to Cd toxicity stress conditions by lowering Cd accumulation, facilitating the formation of disulfide bonds, sustaining intracellular calcium homeostasis, and regenerating the waxy outer layer of the leaf surface. These results showed that applying SeO_3_^2^⁻ at an enhanced level (20 mg L^−1^) had negative impacts on the growth of *B. napus*, while applying 20 mg L^−1^ Se-NPs caused a dose-dependent increase in chlorophyll content [81]. Se treatment increased SOD and glutathione reductase (GR) activity in the rice plant under Cd toxicity stress conditions because lipid peroxidation was reduced [82]. Se treatment elevated CAT and POD activity ROS, antioxidant enzymes, and isozymes of rice plants [83].

Se (10 mg L^−1^), CS (0.1%), and a combination of two concentrations of CS-Se NPs (5 and 10 mg L^−1^) were administered to Moldavian balm (*Dracocephalum moldavica* L.) plants at 0, 2.5, and 5 mg kg^−1^ Cd-stress conditions. The outcomes revealed that the administration of Se and CS-Se NPs might reduce the adverse impacts of Cdstress by improving agronomic qualities, antioxidant enzyme activities, photosynthetic pigments, proline, and phenols, and reducing MDA and H_2_O_2_ [84]. They concluded that a 5 mg L^−1^ concentration might be the most effective strategy. Under 2.5 mg kg^−1^ cadmium toxicity stress, the administration of Se and CS-Se NPs increased the amount of chlorophyll b and carotenoids. Ikram et al. [18] studied the *Allium sativum* L. clove extract-based Se-NPs synthesized at different concentrations (25, 50, 75, and 100 mg L^−1^), which were externally supplied to huanglongbing (HLB)-affected Kinnow mandarin trees. Comparing treated and untreated diseased plants, Se-NPs at 75 mg L^−1^ demonstrated a remarkable enhancement in the levels of chlorophyll a (72.61%), chlorophyll b (65.66%), total chlorophyll (50.62%), carotenoid (64.58%), membrane stability index (66.81%), relative water (70.53%), and increased sugar (65.16%). Significantly, proline content (70.96%), H_2_O_2_ (64.89%), and MDA (66.81%) concentrations decreased when compared to untreated mandarin trees.

The biogenic Se-NPs (200 mg L^−1^) increased heat tolerance in *Chrysanthemum* by increasing the activity of peroxidase and catalase and decreasing polyphenol oxidase [33]. El Badri et al. [37] observed that 150 mol L^−1^ bio Se-NPs enhanced the length of shoots and roots by 8.47 and 24.74%. The enhanced level of Se (bio Se-NPs 150 mol L^−1^) also enhanced the total chlorophyll and chlorophyll a and b. Additionally, proline content significantly increased at conditions of 50, 100, and 150 mol L^−1^ by 21.60, 29.23, and 33.14% (bio Se-NPs), and 86.40, 125.69, and 243.38% (Na_2_SeO_3_), accordingly. In conclusion, it was observed that seedlings treated with bio Se-NPs surpassed Se (IV) in maintaining total leaf K^+^ concentration during salt stress [37]. Cui et al. [85] studied the medicinal plant *Glycyrrhiza uralensis* Fisch. in pot culture experiments under salt stress such as NaCl, SiO_2_ kg^−1^ and found that it enhanced sucrose synthetase (SS) and sucrose phosphate synthetase (SPS) activity.

According to Neysanian et al. [86], tomato plants exposed to foliar sprays of nSe at 0, 3, and 10 mg L^−1^ or equivalent dosages of sodium selenate showed elevated shoot and root biomass at 3 mg L^−1^ but considerably decreased biomass accumulation at 10 mg L^−1^. The BSe/nSe treatments transcriptionally increased the levels of miR172 (3.5-fold) and the bZIP transcription factor (9.7-fold) in leaves. In comparison, the leaves and fruits of the nSe-treated seedlings had an average transcriptional increase of 5.5 times in the carotene isomerase (*CRTISO*) gene.

The effectof nano-priming on the transcription levels of genes of GA biosynthesis, such as *BnGA3ox2*, *BnGA20ox1*, *BnGA20ox2*, *BnGA3ox1*, *BnGA20ox3*, and *BnCPS*, during rape seed germination under salt treatment, were examined. Those genes showed distinct expression levels, notably after being nano-primed by Se-NPs and ZnONPs after the salt stress subjection.

Furthermore, only *BnGA20ox1* showed the least expression among the *BnGA20*-oxidase genes, and nano-priming with Se-NPs and ZnONPs raised their levels of countenance by 61.97% and 110.72% (salt-tolerant variety—Yangyou 9), 36.37% and 65.35% (salt-sensitive variety—Zhongshuang 11), respectively, in comparison to unprimed seeds and hydroprimed seeds at 24 h after sowing [26]. Recent research has focused on the transcriptional responses to nSe in several different plant species, including wheat Heat shock transcription factor A4a, *Melissa officinalis* (Hydroxyphenylpyruvate reductase and rosmarinicacid synthase, pepper (*bZIP* transcription factor), chicory (Hydroxycinnamoyl-CoA shikimate transferase 1, *PAL*, *Dehydration responsive element binding factor-1*, and hydroxycinnamoyl quinate hydroxycinnamoyltransferase genes), and bitter melon (*WRKY1*, *PAL*, and 4-Coumarate:CoA ligase, as reviewed in [86]. Soliman et al. [87] investigated physiological alterations in leaves and their impact on wheat crop yield in salt-affected soils. Se-NPs significantly stimulate water transporter genes in these studies, specifically *P1P1*, *NIP*, and *N1P1*. These genes were stimulated in saline stress and were considerably enhanced when straw biochar (SB) was administered. Wheat exhibited improved carbon absorption and biomass formation in SB and treatments, which might be associated with the activation of water transporter genes [87].

### Influence on Primary and Secondary Metabolites

Plant secondary metabolism in response to NPs is necessary for plant performance, communication, and adaptation. Modifications in plant secondary metabolism brought on by NPs may have an impact on how well plants interact with their environment, which may have an impact on growth and productivity [88]. NPs can modulate plant secondary metabolism by interfering with numerous different signaling pathways. Plants’ initial responses to NPs often include increased levels of ROS, cytoplasmic Ca^2+^, and activation of mitogen-activated protein kinase (MAPK) cascades, substantially like other abiotic stresses. The induction of ROS in response to NP interactions has been evidenced throughout species of plants. The established link among ROS and secondary signaling messengers leads to secondary metabolism transcriptional regulation. The existing connection between secondary signaling molecules and ROS results in transcriptional regulation of secondary metabolisms.

Se may alter the secondary metabolism and biomass in plants, resulting in a higher concentration of certain health-promoting phytocompounds [15]. Battin et al. [89] stated aconcern regardingprimary metabolites; there is a rise in the activity of glutathione peroxidase (GPX), an enzyme whose catalytic cycle includes selenic acid (PSeOH). This combines with glutathione, which acts as a coenzyme in this process, to generate a selenyl-sulfide adduct [89]. Ascorbate peroxidase, catalase, superoxide dismutase, dehydroascorbate reductase, glutathione reductase, and monodehydroascorbate reductase are only a few of the enzymes with an antioxidant capacity that are made more active by selenium treatment [90].

Schiavon et al. [90] examined the impact of two selenium fertilization strategies on levels of anticarcinogenic seleno compounds in radish roots and discovered that the thiols cysteine and glutathione were existent at two-to-three-fold higher levels in Se-treated plants’ roots. Increases in glucoraphanin led to 35% higher levels of total glucosinolate. Se-methyl-SeCys is the only seleno-aminoacid present in Se-treated plants (100 mg kg^−1^ FW in leaves and 33 mg kg^−1^ FW in roots) that was noticed. They found that foliar Se administration in radish (*Raphanus sativus*) altered the synthesizing phenolic chemicals individually in leaves and roots in distinct ways. There are 11 major polyphenols found in leaves; five were flavonols and kaempferol derivatives, while the remaining six of the compounds constituted hydroxycinnamic acids. Despite the fact that sinapic acid, caffeic acid, kaempferol-3-O-arabinoside-7-O-rhamnoside, kaempferol-3-ramnosil glucoside, and kaempferol-3,7-diramnoside, which were explored to increase at particular Se dosages, the other identified phenolics (cumaric acid, kaempferol-7-O-rhamnoside) did not exhibit any increase (kaempherol-3-glucoside, ferulic acid, feruilmalatesinapoil-malate). In the past few years, countless researchers have examined the role of nanoparticles as an elicitor for stimulating the expression of genes that participate in secondary metabolite biosynthesis [91]. Elicitors may induce gene expression to create enzymes that boost the route of secondary metabolites [92]. Existing studies by Hussein et al. [93] and Zahedi et al. [94] have also revealed that Se-NP application promotes plants to generate higher secondary metabolites. Corresponding to this, Ciccolini et al. [95] showed that Se-induced accumulation of phenols and flavonoids in lettuce increased antioxidant effectiveness and stress adaptation. According to Li et al. [96], application of foliar treatment of 5 mg L^−1^ Se-NPs boosted celery’s overall antioxidant capacity by 46.7%, total flavonoids by 50%, particularly apigenin, p-coumaric acid, ferulic acid, luteolin, kaempferol, total phenols (21.4%), and vitamin C (26.7%). The effects of Se-NPs on the biochemical characteristics of cluster beans (*Cyamopsis tetragonoloba*) were studied by Ragavan et al. [22]. In these studies, cluster bean pot culture tests were conducted with varying concentrations of selenium nanoparticles (0, 100, 200, 300, 400, and 500 mg for treatment T_0_ (Control), T_1_, T_2_, T_3_, T_4_, and T_5_), and growth biochemical and yield estimates were performed after 60 days. They observed that T_4_ plants had higher chlorophyll a, b total chlorophyll, carotenoids, anthocyanin, protein, L-proline, free amino acids, and leaf nitrate. Over all, T_4_ has the highest yield of cluster beans among the treatments, while T_0_ has the lowest. Tian et al. [97] analyzed selenium-enriched broccoli and used 25 M Na_2_SeO_4_ to treat two broccoli cultivars. Se supplementation was observed to decrease the formation of total glucosinolates, particularly glucoraphanin, the direct precursor of a strong anti-cancer agent, in broccoli florets and leaves in this investigation. This work demonstrates that after Se supplementation, concentrations of the glucosinolate precursors methionine and phenylalanine, along with the expression of genes associated with glucosinolate production, were significantly lowered. The addition of Se-NPs at a concentration of 100 mg L^−1^ resulted in a significant accumulation of Se in the leaves of barley (*Hordeum vulgare*) cultivated under saline stress, an increase in the amount of aggregate phenolic composites, and a reduction in the content of ROS-mediated cellular membrane injury markers such as MDA, which could affect the metabolism and be the cause of nutrient deficiencies [98]. Soliman et al. [87] found that SB and Se-NP significantly increased phenols, osmolytes, and flavonoid levels in salt-stressed wheat plants, including glycine betaine, proline, and carbohydrates. The enhanced accumulation of phenols and flavonoids improved the antioxidant system, reducing ROS damage to cellular organelles.

## 5. Influence of Se-NPs on Crop Yield

Garca Márquez et al. [99] examined the impact of foliar supplementation of selenium (Na_2_SeO_3_ and Na_2_SeO_4_) in cereal crops at various concentrations between 30 and 300 g ha^−1^. They reported that the selenium application results in the deposition of Se in grains, and the metabolism might be stimulated. In a hydroponic method, the magnification of Se and antioxidants in edible parts of crops such as lettuce, tomato, and strawberry is increased in the form of Na_2_SeO_3_ (0.86 to 5 mg L^−1^) and Na_2_SeO_4_ (0.5 to 18 mg L^−1^), as reviewed in [99]. Se biofortification has beneficial actions on plant metabolism, resulting in the development of molecules with reduced power, which promotes stress tolerance [100], plant growth, and fruits with better nutraceutical qualities. Different crops have improved from various biofortification techniques, which include the exogenous inclusion of ionic forms [15]. Se biofortification of food crops has shown positive outcomes, including a rise in metabolites that improve tolerance from the nutraceutical standpoint.

In wheat roots and shoots, Se-NPs were transported from roots to shoots and promptly absorbed into Se (IV) and organic forms, whereas the smaller diameter bio Se-NPs made by microorganisms may indeed enhance the wheat field to the uptake of Se-NPs. As a result, Se-NPs could therefore be used as a cutting-edge fertilizer in the green revolution [47]. The manufacture and application of nSe as a nutrient and biofortifier has been indicated to be an innovative method since it has been discovered to have superior biocompatibility, chemical stability, quick absorption, and lower toxicity than ionic forms of this element [79,96]. Foliar application of sodium selenate to leaves (up to 5 mg L^−1^) under normal and salt stress circumstances in canola plants dramatically enhanced the plant pods, seed numbers, and seed weight [101].

In pot culture experiments, with and without Si NP foliar supplement, Golubkina et al. [102] evaluated the individual and combined foliar application of chervil plants with potassium iodide (KI, 150 mg L^−1^) and Na_2_SeO_4_ (10 mg L^−1^). In all treatments, nano-Si (14 mg L^−1^) enhanced the shoot biomass compared to control plants: 4.8 times with Si, 2.8 times with KI + Si, 5.6 times with Se + Si, and 4.0 times with KI + Se + Si. Se, KI, and KI + Se treatments had a growth-stimulating impact on chervil shoots and roots, which increased by 2.7, 3.5, and 3.6 times, correspondingly. The foliar application of Na_2_SeO_4_(1 mg L^−1^) in tomatoes considerably delayed the fruit ripening and preserved the fruit quality. Lower ethylene synthesis and respiration rate were recorded with inhibiting ethylene biosynthesis genes such as 1-aminocyclopropane-1-carboxylic acid (ACC) synthase and ACC oxidase [103].

Foliar Se treatments (50 g ha^−1^) carried out on Cowpea (*Vigna unguiculata* L. Walp.) plants that grow in acidic and weathered soil conditions such as in tropical soils increased chlorophyll production and photosynthesis in addition to the antioxidant activities and productivity of the plant [104]. Cd-contaminated soil was used to grow the wheat plants, whereas they accumulated more biomass and had increased antioxidant enzyme function as a result of Se (Na_2_SeO_4_) foliar spraying at various growth stages. While leaf SOD, CAT, POD, and APX are dramatically increased with the supply of Se, lipid peroxidation is reduced [105]. By promoting the activity of glutamate synthase and nitrate reductase in lettuce, the foliar application of Se improved nitrogen metabolism [106]. Se-NP concentrations of 265–530 µM noticeably prompted organogenesis and root system development (40%) in tobacco callus cultures, whereas the form of selenate completely inhibited organogenesis and root system development. During an in vitro tobacco tissue culture (*Nicotiana tabacum*), it was reported that the administration of Se-NPs (265–530 M) enhanced the organogenesis and expansion of the root system [59].

The uniformly sized mature seeds of *Brassica napus* types such asthesalt-tolerant variety (Yangyou 9) and the salt-sensitive variety (Zhongshuang 11) were hydro-primed (HP) in ddH_2_O with 150 mol L^−1^ Se-NPs and 100 mg L^−1^ ZnONPs for 8 h. Se-NPs (150 mol L^−1^) enhanced CAT by 11.51% and 17.31% compared to Hydro priming in Yangyou 9 and Zhongshuang 11, respectively. Se-NPs also increased SOD activity by 3.386% and 3.981%, POD activity by 12.33% and 12.50%, APX activity by 15.78% and 12.20%, and SOD activity by 3.386% and 3.981% [26].

Apart from the Se-NPs, other nanomaterials are excessively incorporated into the environmental system. It is crucial to treat or recycle excess Se-NPs once they have been used in suitable farmlands to prevent environmental problems. In this regard, Padervand [107] worked on the 3-Glycidoxypropyltrimethoxysilane (GPTMS) decorated magnetic core—aluminosilicate shell Na (SiA1)06.XH_2_O/NiFe0_4_) prepared hydrothermally. This method may successfully remove the dangerous lead and cadmium ions from waste fluids. The different types of applications of biosynthesized Se-NPs in plant growth, yield and stress tolerance are shown in Table 2.

## 6. Impact of Se-NPs on Abiotic and Biotic Stress Tolerance

NPs can be employed to minimize the aggregation and toxicity of heavy metals as well as protect plants against environmental effects such as salt or drought stress. NPs can act as a supply of micronutrients, boosting fitness and assisting plants in surviving stressful situations. Exogenous applications of plant-mediated Se-NPs boosted the function of APX, SOD, and CAT enzymes and subsequently stimulated the expression of antioxidant defense-related genes in maize and strawberry plants, enhancing their ability to withstand a/biotic stresses [129]. Meanwhile, according to a different study, exogenous foliar spray of Se-NPs reduced H_2_O_2_ and lipid peroxidation levels while increasing the activity of anti-oxidant enzymes, including peroxidase and SOD, in strawberry plants under salt stress [94].

Several antioxidant enzymes, namely SOD, APX, and CAT, increased drought tolerance in tomatoes treated with 40 M of selenium, according to Rady et al. [75]. APX accelerated drought tolerance by 44%, SOD increased drought tolerance by 56%, and CAT enhanced drought tolerance by 57%. The foliar mode administration of groundnut plants with nSe (40 mg L^−1^) for 45 days results in enhanced unsaturated fatty acids and antioxidant capability, which greatly increases plant growth [93]. Pomegranate plants under drought stress had better growth and yield owing to the Se-NP (10 nm) concentrations, and apart from that Se-NPs (10 nm) also improve the activity of photosynthetic pigments, nutritional status, antioxidant activity, and total phenolic content levels during drought conditions [94]. The Se-NPs are biologically synthesized by reduction with leaf extract obtained from the barley (*Hordeum vulgare* L.) plant used to lessen the harmful effects of saline stress. One of the main abiotic factors that impact plant crops is salinity, which affects 30% of the world’s irrigated agriculture and 7% of rainfed agriculture, ultimately resulting in a 65% loss in agricultural productivity. P^3+^ and Mg^2+^ concentrations in canola plants under control and saline treatments respond positively to Se treatment at 5 mg L^−1^ [101]. The growth and yield metrics of strawberry plants grown under normal and drought stress conditions (30, 60, and 100% foot-candles) were increased by spraying solutions of nanoparticles of SiO_2_, Se, and Se/SiO_2_ (50 and 100 mg L^−1^). The benefits of nanoparticles of SiO_2_, Se, and Se/SiO_2_ (SiO_2_-NPs, Se-NPs, and Se/SiO_2_-NPs) were shown in moderating the adverse effects of drought on the growth and yield of strawberry plants [130].

During high and low-temperature stress, a lower dosage of Se (2.5 µM) and nSe (1 µM) can increase plant growth performance more significantly than a high proportion of Se/N-Se (2.5 µM) [109]. The administration of 20 g Se to the *Brassica napus* L. plants resulted in a considerable increase in the dry weight of vegetative portions and pods and seeds [131]. At the same time, 20 ppm Se-supplied *B. napus L.* plants exhibited superior photosynthetic rate and protein content than control plants [131]. Dong et al. [132] reported that *Lycium chinense* L. leaf contents such as chlorophyll, chlorogenic acid, and carotenoids were increased substantially by 200–400% when selenium (10–50 ppm Se) was applied. 

The supply of nano-SiO_2_ (10 mg L^−1^) to cotton soil did not affect plant Si concentration, whereas it substantially enhanced the IAA production [133]. Se-NPs might sustain Se beneficial actions at lesser concentrations to protect plants from metal toxicity via a reduction in oxidative stress, decreased metal uptake, and translocation [134]. According to Wu et al. [105], Se boosted the enzymatic activities of SOD and CAT levels in the roots and leaves of Chinese cabbage under Cd-stress conditions. The desirable features and distinctive bioactivities of Se-NPs, which have drawn interest in agricultural applications [135], have principally shown considerable economic benefits in helping plants to withstand heavy metal or other abiotic stress situations [136].

Gao et al. [137] reported that Se prevents hazardous metals from being absorbed and transported from the roots to the top plant parts, including shoots, leaves, and grain. Se-NPs(10–40 nm) were used to study the toxicity, physiological, and biological impacts of heat stress on *Sorghum bicolor* (L.) Moench [66]. Enhanced pollen germination rates and larger amounts of unsaturated phospholipids were accomplished, which also increased seed yield under heat stress. Administration of Se-NPs raised the percent seed yield (14%), seed set (19%), and pollen germination (26%). Selenium boosted the CAT, POD, and SOD activities in the drought-stressed cucumber roots [74]. This implies that the Se-NPs increased proline production by increasing nitrate reductase activity, which is necessary for proline synthesis. Se supplementation increases pectin and hemicellulose content as well as cell wall thickness, which improves harmful metal binding by the cell wall [65]. Babajani et al. [138] and Soleymanzadeh et al. [139] reported that an important mechanism that enhances plant defense against abiotic stress conditions is the Se-mediated stimulation of the antioxidant system. A study by Morales-Espinoza [124] investigated the impact of Se-NPs on tomato antioxidant responses, plant growth, and fruit quality under NaCl stress. The effects of four dosages of Se-NPs (1, 5, 10, and 20 mg L^−1^) were studied under sodium chloride stress-treated and control tomato plants. The aerial biomass and fruit mass responded favorably to the treatment (10 mg L^−1^ of Se-NPs + NaCl). Treatment with 20 mg L^−1^ of Se-NPs with NaCl resulted in the greatest levels of total chlorophyll and chlorophylls a and b. Compared to the other treatments, 10 mg L^−1^ of Se-NPs + NaCl and 20 mg L^−1^ of Se-NPs + NaCl were preferable [124]. Ikram et al. [18] investigated the effects of Se-NPs produced from *Allium sativum* L. gloves extract against drought stress in wheat plants. Remarkably, it was found that wheat plants under drought stress exhibited enhanced agronomic attributes (number of leaves per plant, shoot length, root length, and plant height) when exposed to plant-mediated Se-NPs at a concentration of 30 mg L^−1^.

The lesser level of Se-NPs promotes the development of *A. niger*, which may be related to trace elements’ role in microbial growth promotion. Low levels of Se also enhance the development and yield of ryegrass, *Brassica*, soybean, and potato plants. According to earlier findings, applying Se-NPs at a lesser level coupled with *Trichoderma* as a seed treatment improves plant growth characteristics, as reviewed in [36]. Plant pathogen *Sclerospora graminicola* affects maize and pearl millet crops. According to a previous study, *S. graminicola’s* growth, spore viability, and sporulation are inhibited by biosynthesized Se-NPs [36]. The development and photosynthetic ability of *Nicotiana tabacum* were shown to be negatively impacted by the supplementation of selenate at 10 mg L^−1^. However, no adverse effects were seen when selenate 1 mg L^−1^ was applied at higher concentrations (100 mg L^−1^), according to Zsiros et al. [140]. Inductively coupled plasma mass spectrometry (ICP-MS) determines strawberry plants’nSe absorption and accumulation rates under salt conditions [141]. Effects of nSe (10 and 100 µM) on strawberry plants subjected to salt stress were assessed in terms of photosynthetic performance, ion homeostasis, antioxidant system, and phenylpropanoids level and water-splitting complex (Fv/Fo), and the results revealed that the above parameters were improved as a result of the nSe treatment at 10 µM. In strawberry plants under drought stress, Se and SiO_2_ NPs had enhanced photosynthetic pigments compared to many other treatments. Additionally, they showed that 100 mg L^−1^ Se/SiO_2_ NPs enhanced the relative water content, water usage efficiency, and membrane stability [130].

Matraszek-Gawron [142] assessed the effects of Ni (5 and 10 mM)-subjected lettuce plants receiving 2 or 6 mM of various types of Se (selenite IV and selenate VI administered as Na_2_SeO_3_ or Na_2_SeO_4_). Se administration can reduce the absorption of metal by roots and transformation to shoots, a critical metal/metalloid stress tolerance mechanism [51]. Lower Se concentrations can activate the production of auxin, which imposes root architectural modification and enhanced root development, resulting in lower metal uptake [143]. Se nanoparticle treatment of tomato plants increased enzymatic antioxidants in tomatoes and indicated that Se-NPs activate the antioxidant system more effectively than selenate. Compared to control plants, Se and plant growth-promoting rhizobacteria (PGPR) are highly active at enhancing the antioxidant system, which helps to explain the elevated activity of antioxidant enzymes (APX, GPX, and CAT) in pretreated plants [86].

Mohammadhassan et al. [144] demonstrated that the NanoFe (10 mg L^−1^) and NanoSe (1 mg L^−1^) as the most appropriate treatment for the biosynthesis of maximum antioxidant activity (11,974 µg mL^−1^) in in vitro studies on *Asparagus officinalis*. Se-NPs are particularly effective against many diseases, including the downy mildew in pearl millet caused by *Sclerospora graminicola*, as reviewed in [99], tomato leaf blight induced by *Alternaria alternata*, and late blight of tomatoes caused by *Phytopthora infestans*. According to El-Saadony et al. [127], green-produced Se-NPs are highly efficient towards *Fusarium* species-caused root rot and crown diseases in wheat crops.

Biofortification is a process that tries to improve the Se level in edible plant parts to prevent Se deficiency in humans and cattle, and Se-NPs could be employed for Se biofortification. Investigations on *Nicotiana tabacum* and *Allium sativum* have demonstrated that Se-NPs are less damaging to plants than ionic Se salts (SeO_4_^2−^ and SeO_3_^2−^) [96]. Application of 50 M selenite (Se IV) to *B. juncea* managed to improve germination by increasing seedling vigor and germination rate as well as growth by decreasing the As-based (As III) harmful actions in shoot and root length, fresh and dry mass of the plant, and root/shoot ratio by about 9, 12, 5, and 9%, respectively [145]. The Se (8 mM) administration boosted Ca and K levels and salt stress resistance in bread wheat exposed to salt stress [146]. Se-NPs have been linked to significantly improved Cd-mediated oxidative damage in *Brassica napus* [81]. When selenium was applied to quinoa plants in limited concentrations (2.5 and 5 mg L^−1^), the plant’s growth parameters and antioxidative enzymes all significantly increased under diverse abiotic stressors [147]. In particular, Se-NP treatment boosted the proline level in control and Cd-stressed plants [148].

In a previous study [149], Se-NPs (1 mg L^−1^), various bacteria (*Bacillus cereus,* and *Pseudomonas fluorescens)*, and a combination of both were used to prime foxmillet seeds before they were planted in pots and subjected to salinity stress (0, 100, and 200 mM) after two weeks. The results revealed that the ability of a plant to handle salinity stress could be improved by soaking millet seeds in Se-NP solution, improving photosynthetic pigments, lowering oxidative stress, preserving the cell membrane and suitable solutes, and lowering sodium uptake. When PGPRs bacteria develop, these effects are accelerated. Secondary stresses, including oxidative stress, which is detrimental to plant cells due to steep ROS, osmotic disparity, and water deficits, frequently occur along with salinity-induced osmotic stress, leading to ion toxicity in stressed plants, as reviewed in [37]. Se-biofortified tomato fruits were produced when selenate was applied as a foliar spray (0, 2, or 20 mg Se); as a result, the selenate level is sufficiently low and is not adverse to human health. Schiavon et al. [150] illustrated that Se-biofortified fruits had higher amounts of antioxidants.

Commercially available Se-NPs (80 to 100 nm) enhanced the growth recovery of wheat plants subjected to salt stress more effectively than soybean straw biochar (SB), according to Soliman et al. [87]. Their results depicted that the salt-stressed plants applied with SB and biochar + led to 25%, 33%, and 44% more root dry weight, respectively. Designing a feasible and ecologically sustainable method for producing selenium nanoparticles has gradually gained more attention [151]. Se-NPs’ ability to adsorb substances makes them useful for the remediation of soil and water contaminated with metals and heavy metals. Regarding their use as medicinal agents and food additives, SeNPs have drawn particular attention [152].

## 7. Conclusions

Selenium is a crucial microelement vital for the growth and development of various life forms, including plants. Nanomaterials are potentially active compared to their bulk form due to their large surface area and tiny size. This review presented the various types of Se-NP synthesis approaches, optimal conditions for synthesis, and their characterization methods. Se-NPs’ influence on the plants’ growth and physiology was discussed in detail. Their impact on the production of primary and secondary metabolites and their functions concerning stress tolerance were provided. This review will help researchers to understand the mechanistic functions of Se-NPs in the growth and yield, nutraceutical quality, and metal, biotic, and abiotic stress tolerance in plants, and will provide insight for using Se-NPs in agriculture. Detailed molecular research on Se-NPs’ uptake, translocation, distribution, and how they influence growth and stress tolerance in plants is warranted to increase their application in sustainable modern agriculture. Additionally, their utilization in nano-agrochemicals is also essential in future modern agricultural applications.

## Figures and Tables

**Table 2:** The dose, mode of application, and the impact of biosynthesized Se-NPs onplant growth and development.

Plant Species	Se-NPsSource (Selenium and Their Combination)	Dosage/Size	Mode of Application	Impact of Se-NPs	Biochemical/ Molecular Function	References
*Lactuca sativa*	Na_2_SeO_3_ and SeO_3_	50 mg L^−1^	Foliar spray	Plant growth	1. Chlorophyll content was elevated at the early stages of plant germination. 2. Senescence prevention	[108]
*Brassica napus*	Na_2_SeO_4_	2.5 and 5.0 mg L^−1^	Foliar spray	Plant growth and yield	Increased in yield, shoot length, and number of leaves plant^−1^ under salt stress conditions	[101]
*Solanum lycopersicum*	N-Se, Na_2_SeO_4_	1 µM and 2.5 µM	Foliar spray	Plant growth at low and high-temperature conditions	Improved 27.5% green pigments content in hydroponic culture	[109]
*Triticum aestivum*	Se	7.06 μM	Foliar spray	Plant growth	Maintaining higher growth, fresh and dry matter content	[110]
*Brassica juncea*	Na_2_SeO_4_	10 µM	Foliar spray	Plant growth improvement	Enhancing growth and photosynthesis efficacy	[111]
*Nicotiana tabacum*	Na_2_SeO_3_	6 mg kg^−1^	Foliar spray	Plant growth improvement	Improved plant growth by uplifting plant photosynthesis	[112]
*S. lycopersicum*	Na_2_SeO_4_	10 to 20 mg kg^−1^	Directly to peat	Plant growth improvement	Increased vitamin A content in tomato fruit	[113]
*S. lycopersicum*	Na_2_SeO_4_	1 mg L^−1^	Foliar	Improved plant growth and disease management	1. Increased antioxidant activity. 2. Control of gray mold rot infection	[114]
*Raphanus sativus* cv. Saxa	Se	5 mg	Foliar spray	Improved plant growth	Increasing the polyphenol content (flavanols, kaempferol derivative, and hydroxycinnamic acids) up to 10% higher than the control.	[90]
	Se	10 or 20µM	Foliar spray	Improved plant growth	1. Enhanced in Glucosinolates, 2. Dimeric-4 mercaptobutyl (DMB-GLS) concentration	
	Se	40 µM	Foliar spray	Improved plant growth	In plant root systems, the stimulation of high-affinity sulfate transporter genes (*Sultr1;1* and *Sultr1;2*)	
*S. lycopersicum*	Na_2_SeO_4_	1 mg L^−1^	Foliar pre-treatment	Improved plant growth	1. Delay in tomato fruit 2. Inhibition of ethylene biosynthetic genes—ACC synthase	[103]
*Fragaria × ananassa*	Na_2_SeO_4_	1.9 and 19 mg L^−1^	Nutrient solution	Plant growth and fruit yield	Growth regulator’s upregulation, biomass, and nutraceutical quality	[115]
*Ocimum basilicum*	Na_2_SeO_4_	4, 8, and 12 mg L^−1^	Nutrient solution	Plant growth	Biofortification (Se enrichment in the leaves)	[116]
*Oryza sativa*	Na_2_SeO_3_ and Na_2_SeO_4_	120–300 g ha^−1^	Foliar	Improved yield	Enhanced Se content in the rice grains	[117]
*Cyamopsis tetragonoloba*	Se-NPs	400 mg	Foliar spray	Enhancing the growth yield	Enhancing the biochemical activity	[22]
*Fragaria × ananassa*	Se	100 µM	Nutrient solution	Enhancing the growth	Enhanced the accumulation of anthocyanins	[115]
*L. sativa*	SeO_2_	5 mg L^−1^	Nutrient solution	Enhancing the growth and biomass	Accumulation of 24 mg Kg^−1^ of Se in leaves (Dry Weight)	[118]
*Citrus aurantifolia*	Se-NPs	50 mg L^−1^	Imbibition of seeds	Plant growth improvement	Plant growth was improved	[119]
*T. aestivum*	Se-NPs	5 µM	Imbibition	Plant growth improvement	Enhance the root aquaporins	[47]
*Daucus carota*	Na_2_SeO_4_	1 mg L^−1^	Foliar apply	Enhancing the yield	Increased yield, decreased fruit ripening	[120]
*S. lycopersicum*	Na_2_SeO_4_	1 and 1.5 L^−1^	Hydroponics	Yield improvement	Delayed postharvest ripening	[121]
*T. aestivum*	Na_2_SeO_4_	10 g ha^−1^	Soil application	Yield improvement	50% accumulation of Se in grains	[122]
*T. aestivum*	Na_2_SeO_4_ + surfactant	120 g ha^−1^	Foliar apply	Enhancing the growth and biomass	1. Increased production by 48% and biomass by 30% 2. Increasing the grain weight (DW)	[123]
*Arachis hypogaea*	nSe	40 mg L^−1^	Foliar	Enhanced plant growth	Improved antioxidant potential	[93]
*S. lycopersicum*	nSe	10 mg L^−1^	Foliar	Plant growth improvement	1. Induce the salinity tolerance of growth 2. Enhanced antioxidant enzyme activity	[124]
*Lubia*	nSe	1.18 mg L^−1^	Imbibition of seeds	Plant growth improvement	The total proteins, sugars, and increased seedling enzyme activity (α, β amylase, and protease)	[125]
*Coriandrum sativum*	nSe	25 and 50 mg L^−1^	Foliar + surfactant tween 80–0.005	Plant growth improvement	Ascorbic acid content was improved.	[71]
*Vicia faba*	nSe	10 and 20 mg L^−1^	Imbibition of seeds	Yield improvement	The cytotoxicity activity	[126]
(*Fragaria* × *ananassa*)	Se-NPs	10 and 20 mg L^−1^	Foliar spray	Yield improvement	Enhanced organic acids and sugars content	[94]
*Brassica chinensis*	SeO_3_^2−^	10 μM	Hydroponic	Plant growth improvement	Enhanced antioxidant activity	[80]
*Vigna unguiculata*	Se-NPs	6.25 µM	Foliar applications	Plant growth improvement	Increased the level of Indole Acetic Acid (IAA), Gibberellic Acid, and Cytokinins	[25]
*Apium graveolens*	SeNPs	5 mg L^−1^, 50–78 nm	Foliar spray (Once in 10 days, 3 times application	Plant growth improvement	Increased primary and secondary metabolites	[96]
*T. aestivum*	Bio Se-NPs	100 µg mL^−1^	Mixture with the soil at the rate of 5% (*v*/*w*).)	Plant germination and yield improvement	Improvement of plant growth and 5–40% enhancement of the grain quantity and quality	[127]
*Dracocephalum moldavicum*	Cs–Se NPs	5 mg L^−1^	Exogenously applied	Plant growth and yield improvement	Enhance the improvement of the agronomic traits	[84]
*Momordica charantia*	Cs–Se NPs	10, and 20 mg L^−1^	Foliar spray	Plant growth enhancement	Increasing antioxidant enzyme activity, proline, and relative water content.	[128]
*Hordeum vulgare*	Se-NPs	100 mg L^−1^	Foliar mode	Enhancing plant growth	1. Increase in phenolic composites under saline conditions. 2. Decrease in ROS-mediated cellular membrane harm markers	[98]
*H. vulgare*	Se-NPs	4.65 g mL^−1^	Dosage	Improvement of seedling growth	Greatest seed germination percentage	[68]
*Citrus nobilis × Citrus deliciosa*	Se-NPs	25, 50, 75, and 100 mg L^−1^	Bio-Fabrication	Improvement of yield	Improving the content of carotenoids, chlorophyll, flavonoid, and soluble sugar)	[27]
*Brassica napus*	Bio Se-NPs	150 µmol L^−1^	Exogenously applied	Improvement in growth	Increased the shoot and root length under salt stress conditions.	[37]
*T. aestivum*	Se-NPs	30 ppm (once a week)	Soybean straw biochar mixed with soil media	Improved growth	Significantly increased PSII efficiency under salt treatments	[87]

## Data Availability

The data presented in the present study are available in the article.
